# Current Surgical Perspective on the Prognosis of Small-Cell Lung Cancer

**DOI:** 10.3390/diagnostics15212704

**Published:** 2025-10-25

**Authors:** Hüseyin Fatih Sezer

**Affiliations:** Department of Thoracic Surgery, Kocaeli University Medical Faculty, İzmit 41380, Kocaeli, Türkiye; hfs.hfs@gmail.com; Tel.: +90-262-3037500

**Keywords:** small cell lung cancer, prognosis, survival, lung surgery

## Abstract

Small-cell lung cancer (SCLC) is a highly aggressive neuroendocrine tumour that can metastasise early, may show resistance to systemic treatment, and has a poor prognosis. The use of tobacco products is closely related to the duration of their use, and approximately 95% of those diagnosed have a history of smoking. No satisfactory progress has been made in the prognosis with current treatment methods up to the present day. The treatment approach has traditionally involved long-term chemotherapy (CT) and radiotherapy (RT), and recent literature has focused on immunotherapy and genetic advancements. Surgery can only be performed in cases detected at an early stage. Although both chemotherapy and radiotherapy are indispensable options for most patients, their impact on prognosis and survival is limited. Although promising developments are expected in immunotherapy, its impact on survival is still very limited, lasting only about 2 months. In patients undergoing surgical resection as part of their treatment, overall survival (OS) ranges from 34 to 69 months. OS for 1 year is 84.8–93.8%, for 3 years is 60–71.2%, and for 5 years is 51.1–63.8%. The five-year survival rates are reported as follows: stage I 31–63.8%, stage II 25–65.5%, stage III 15–27.8%, and stage IV 0%. In this study, the prognosis and factors affecting prognosis in SCLC were investigated in light of current literature from a surgical perspective, and predictions were attempted to be made to lay the groundwork for personalised treatment approaches. Compared to non-small-cell lung cancer, the number of studies is quite limited. Most of the surgical case series were conducted in the past, retrospectively, and involved a small number of patients. Advances in immunotherapy are promising. In the early stages, resection and subsequent chemotherapy may be the main treatment.

## 1. Introduction

**Figure 1:**
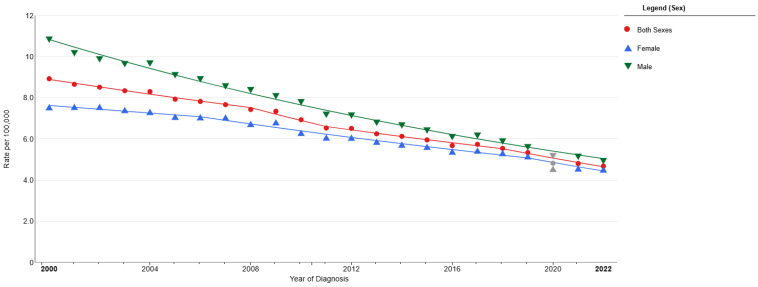
Incidence after the year 2000 in both sexes. National Cancer Institute (https://seer.cancer.gov) [20].

Small-cell lung cancer (SCLC) is a highly aggressive neuroendocrine tumour that can metastasise early, may show resistance to systemic treatment, and has a poor prognosis [1,2,3,4,5,6,7]. It is estimated that there are 250,000 new cases worldwide each year, with 226,650 new cases and 124,730 deaths in the USA in 2025 [8,9]. The use of tobacco products is closely related to the duration of their use, and approximately 95% of those diagnosed have a history of smoking [6,8,10,11]. So much so that initially, the proportion within lung cancers was 17–20%, but due to changes in smoking rates, this has now decreased to 13–15% [1,12,13,14,15,16,17,18,19] (Figure 1). Although SCLC is more commonly observed in males, the observed rates between the two sexes have become more similar recently due to the increasing use of tobacco products in female sex [2,7,8,19] (Figure 1).

Primarily the stage, as well as general medical condition, medical history, gender, race, and treatment summary can be listed as factors that may affect the prognosis [2,13,21]. As can be understood from here, it would be more beneficial to consider personal characteristics rather than generalisations when predicting prognosis. The treatment approach has traditionally involved long-term chemotherapy (CT) and radiotherapy (RT), and recent literature has focused on immunotherapy and genetic advancements. Surgery, however, can only be applied in cases detected at an early stage; considering that 70–80% of patients are diagnosed at an advanced stage, only a limited group are eligible for surgery [2,6,12,22,23]. Unfortunately, no satisfactory progress has been made in the prognosis with current treatment methods up to the present day [24].

Due to the limited number of patients diagnosed at an early stage and the prejudices that surgical treatment will have a limited impact on prognosis, the number of patients who have undergone surgery, and consequently the literature related to surgical prognosis, is quite limited. In this study, the prognosis and factors affecting prognosis in SCLC were investigated in light of current literature from a surgical perspective, and predictions were attempted to be made to lay the groundwork for personalised treatment approaches.

## 2. Staging System

The staging that forms the basis of clinical use was established by the Veterans’ Administration Lung Study Group (VALSG) (1957), and tumours limited to one hemithorax (treatable with a single radiotherapy field) were classified as limited stage (LD), while others were classified as extensive stage (ED). Subsequently, the International Association for the Study of Lung Cancer (IASLC) (2009) recommended the use of the TNM staging system [25]. However, the idea that the staging cannot be effectively determined, or the fact that previous studies have used the first staging, has left the issue of which staging should be used open to discussion [1,2,6,12,26]. From a practical clinical perspective, roughly speaking, when we synchronise both systems, patients with limited (Stage 1-2-3) and radiotherapy-eligible hilar mediastinal lymph nodes in a single hemithorax are limited; others are considered extensive (Stage 4), and it is observed that TNM classification is more frequently used in surgical decisions [9,11,12,14,18,27,28]. Recent studies on the subject of stage are focusing on subclassifications based on genetic and molecular material [26,27,29,30,31]. SCLC is classified into four main subgroups based on specific biomarkers or molecular factors (transcription factor-based): ASCL1 (SCLC-A), NEUROD1 (SCLC-N), POU2F3 (SCLC-P), and YAP1 (SCLC-Y) [29,30,31]. While the dominant subtypes of ASCL1 and NEUROD1 are generally related to neuroendocrine markers, the YAP1 and POU2F3 subtypes are not as closely associated with neuroendocrine factors and are more involved in interactions with the immune microenvironment [31]. The neuroendocrine characteristics of the tumour play a decisive role in its response to treatment and prognosis. For example, being YAP1 positive is a better prognostic factor. What is expected from these biomarkers is that they will play an important role in the development of personalised treatment strategies in the future for SCLC, a heterogeneous disease.

## 3. Small-Cell Lung Cancer Prognosis and Survival

The variable reasons for the prognosis are that the disease’s clinical presentation is influenced by multifactorial parameters, and the response to treatment is not homogeneous. The most important prognostic factor is the stage. Approximately 65–70% of patients are diagnosed at an advanced stage [10], and for patients at this stage, the median survival generally does not exceed 12–14 months. However, in limited-stage patients, treatment applications (such as chemoradiotherapy, cranial irradiation, etc.) can extend survival up to 18–24 months [32,33]. Besides the stage, important epidemiological parameters include the patient’s overall performance status and medical history, age, male gender, and smoking habits. The patient’s overall performance status is one of the most important prognostic indicators. Those with a poor performance status have a lower quality of life and a significantly shortened lifespan, whereas those with a good performance status have a better quality of life, and both treatment tolerance and survival time are notably improved [5,34]. It is expected that young patients will respond better to treatment, while in older patients, comorbidities will increase with age and the prognosis will be worse due to treatment-related toxicities. Although some studies have considered male gender to be a poor prognostic factor and female gender to be a good one, the effect of gender on prognosis remains a debated issue [15,18]. For example, some authors think that women with comorbidities are at higher risk [2,22]. The reason for this situation may be related to differences in tumour biology and smoking habits in women. Smoking is a negative factor in SCLC patients [34]. Since cigarette use causes multisystem effects, it is important both in terms of survival and prognosis. It is quite rare to encounter a patient with SCLC who smokes. It is generally reported that this group of patients is diagnosed at a younger age and that their survival can be relatively longer compared to smokers [15]. Additionally, it has been reported that in individuals who do not smoke at all and are diagnosed with SCLC, the survival rates are numerically lower, but this difference is not statistically significant in multivariate analysis [15]. The effectiveness of treatment methods, the extent of the performed surgery, lymph node metastasis, recurrence, presence of pleural effusion, weight loss of more than 10%, elevated LDH levels, a neutrophil/lymphocyte ratio of 4 or higher, and electrolyte disturbances such as hyponatraemia can also be considered as additional factors affecting prognosis [5,9,13,21,34,35,36,37]. Malnutrition shortens survival by impairing immune response and treatment tolerance. In prognosis estimation, histological or molecular features that are accepted by all sectors have not yet been identified [10]. Therefore, especially the stage, performance status, and age are important factors to consider in determining the treatment strategy.

The areas most frequently affected by metastasis are the contralateral lung, brain, liver, adrenal glands, and bones [2]. Initially, cranial metastases are observed in 10–18% and generally in 40–60% [1,2,12]. As expected, a single metastasis has a better prognosis compared to multiple metastases, while liver and brain metastases have a poor prognosis [22,38]. Within two years after treatment, approximately 75% of locally advanced disease and over 90% of metastatic disease experience recurrence or progression [2,19]. Despite treatment processes, recurrence is observed in less than 10% of patients within two years [9].

The survival time in untreated widespread disease is approximately 2–4 months [5,9,21]. Between 1973 and 2002, the 2-year survival rate in the limited stage was reported as 15–22%, while in the extensive stage it was 3.4–5.6% [6]. When all stages of patients are considered, the five-year survival rate was initially 4.3%, but at the beginning of the 2000s, this rate increased to 6.3% [1]. In more recent publications, the median survival is reported as 14–24 months in limited stage, 6–12 months in extensive stage, and an average survival of 13 months [7,9,17,34,38]. The 2-year survival rate is reported as 30–40%, the 3-year survival rate is 56.5% (limited stage)—17.6% (advanced stage), and the 5-year survival rate is 0–47% [5,7,9,11,12,14,21,22,23,26,29,30,32,34,39,40,41,42,43,44]. Heterogeneous results among the studies are due to treatment options and universal change. From a traditional perspective, the 5-year survival rate in the limited stage (LD) is 22.8%, while in the advanced stage (ED) it is below 2% [19]. In terms of TNM staging, the survival times are as follows: stage 1a 60 months, 1b 43 months, 2a 34 months, 2b 18 months, 3a 14 months, 3b 10 months, 46 months [11].

Factors affecting survival in the treatment process include concurrent chemoradiotherapy, early radiotherapy, and prophylactic cranial irradiation [23,32,43]. In patients who are at stage 1 and have not undergone surgical treatment, the two-year survival rate is below 5%, whereas in those who have undergone surgery, this rate can reach up to 30% [45]. In stage 2 patients, the median survival time with a combination of surgery and chemotherapy is 24 months, whereas for patients receiving only chemotherapy or radiotherapy, this period is 13–16 months [45]. In Stage 3 patients, the median survival with combined therapy is 20 months (previously 15 months), while in Stage 4 patients, the group receiving only chemotherapy has a median survival of 8 months, whereas with combined therapy it is 12 months [45]. The 5-year survival rate in early-stage patients who undergo surgery combined with chemotherapy is 30–58% [46]. In recent studies, the 5-year survival rate for patients who did not undergo surgery within a limited group between 2018 and 2022 is reported as 53.9% [19].

One of the most significant challenges in SCLC treatment is undoubtedly the rapid resistance that develops against therapy. It is believed that molecular heterogeneity, immunosuppressive colonies in the tumour microenvironment, and differences in DNA repair mechanisms play a role in the formation of this resistance [39,47]. The prognosis of patients with developing resistance generally progresses, and survival with second-line treatments is usually expected to be around 6 months [44].

## 4. The Role of Surgery in Treatment and the Impact of Surgical Treatment on Survival and Prognosis

Since the majority of patients are in an advanced stage at the time of diagnosis, the treatment processes are primarily shaped by medical treatment, radiotherapy, and conservative approaches [1,2,38]. Although both chemotherapy and radiotherapy are indispensable options for most patients, their impact on prognosis and survival is limited [6,9,11,17,18,19,24,29,33,48]. So much so that the two-year survival rate in patients resistant to chemotherapy is below 10% [1]. Chemotherapy treatment is also applied in addition to surgery in patients who have undergone early-stage resection. Treatment models have remained the same for a long time until the developments in immunotherapy. Although promising developments are expected in immunotherapy, its impact on survival is still very limited, lasting only about 2 months [1,2,4,17,24,27,39,44,47,48,49].

In patients diagnosed at an appropriate stage and considered for surgical treatment, the primary selection criteria include tests that demonstrate respiratory function status, cardiac function status, and blood values. The patient’s respiratory reserve is assessed using spirometry and diffusing capacity tests. The patient’s respiratory parameters must be capable of providing single-lung ventilation during the operation and sufficient to maintain the patient’s vital functions after the operation. The ECG and echocardiography tests performed to assess cardiac functions evaluate whether there are any operable arrhythmias and assess pulmonary hypertension. Surgical treatment options are avoided in patients with high average pulmonary artery pressure. In addition to haemogram, coagulometric, and basic biochemistry tests, necessary laboratory tests are also performed considering the patient’s medical history.

Surgical treatment can only be applied in limited stages, which corresponds to less than 5% of patients (T1-2 N0 M0), and among lung cancer cases, it is approximately 1.53% [1,9,12,18,46,50] (Table 1). A diagnosis is often made incidentally during surgery for most of these patients [22,51]. In cases operated on due to solitary nodules, SCLC is detected in 4–12% of the cases [52]. Before the operation, the tumour’s T stage, size, and resectability are assessed using thoracic tomography (CT), while PET-CT can determine the tumour’s stage, activity, the status of mediastinal lymph nodes, and whether there are distant organ metastases. Another function of PET-CT is to serve as a guide for the localisation where tissue sampling will be performed during histopathological diagnosis. Histopathological diagnosis can be obtained through transbronchial and transthoracic biopsies prior to the operation. Additionally, diagnostic samples are obtained to evaluate the presence of metastasis in mediastinal lymph nodes using EBUS or occasionally EUS.

If we consider the last 30 years, one of the notable studies related to surgical treatment is a prospective study conducted by Lad et al. in 1994 regarding the role of surgery, but no difference was found in survival [11]. Results from this and similar studies have caused bias among surgeons, leading to less frequent application of surgery in SCLC cases. However, in subsequent studies [7,19,59] especially in patients who underwent surgery at an early stage, the significantly better survival outcomes have somewhat broken this prejudice.

The quality of life (QoL) in SCLC patients is significantly lower compared to other malignancies. The aims of surgical treatment should include not only controlling the disease but also improving QoL [61]. Patients with SCLC have a low QoL due to the nature of the disease, and there is significant impairment in their physical and emotional functioning [62]. Although there is a significant decline in QoL in the first 3–6 months, it is possible to have a good quality of life despite the deteriorated physical functionality in the later stages [62]. QoL after surgical treatment is superior to physiotherapy in terms of physical, social, and psychological functionality, but it is disadvantageous in some situations such as pain and breathing difficulties. The role of supportive care is important in improving quality of life. This issue has physical, social, and psychological dimensions and often requires a multidisciplinary approach. Supportive care should begin with education before the operation, and after surgery, it can include nutritional support, managing secretions and pain, early mobilisation, and physiotherapy. The recovery process is accelerated with supportive care, ensuring a faster improvement in respiratory capacity, reducing the rate of complications, improving the prognosis, and prolonging expected survival.

### 4.1. Results Related to Surgical Application

Ideally, surgical treatment involves lobectomy and lymph node excision, followed by chemotherapy [8,26]. Anatomical resections have a significant advantage in terms of survival compared to non-anatomical resections [45]. There are only a few studies reporting the effectiveness of lymph node sampling in surgical applications for SCLC patients. Sampling more lymph nodes in two studies has been reported to prolong expected survival [60,63]. However, another study reports that additional nodal sampling may not directly improve survival, it does significantly increase the change of upstaging [64].

Another issue is that the clinical and pathological stages can differ significantly. In some studies, it has been reported that pathological staging is significantly more advanced than clinical staging, and therefore, some have recommended that the case should be considered as a more advanced stage when making surgical decisions [21]. For these reasons, mediastinal lymph node sampling is recommended as much as possible before the operation [65].

### 4.2. Surgical Survival-Recurrence Outcomes

**Figure 2:**
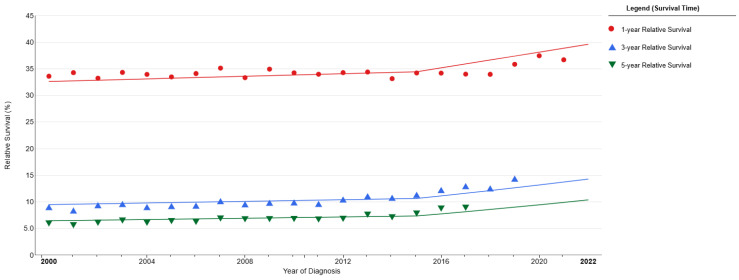
1-3-5 year survival rates. National Cancer Institute (https://seer.cancer.gov) [20].

**Figure 3:**
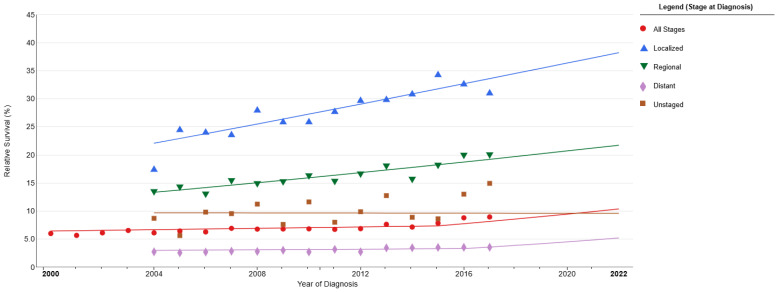
Survival according to stage. National Cancer Institute (https://seer.cancer.gov) [20].

In patients undergoing surgical resection as part of their treatment, overall survival (OS) ranges from 34 to 69 months [50,51,54,60]. OS for 1 year is 84.8–93.8%, for 3 years is 60–71.2%, and for 5 years is 51.1–63.8% [35,60]. The five-year survival rates are reported as follows: stage I 31–63.8%, stage II 25–65.5%, stage III 15–27.8%, and stage IV 0% [2,12,45,50,54,60,66,67]. (Figure 2 and Figure 3) In N0-N1-N2 lymph node metastases, overall survival (OS) was 120-28-40 months, with 2-year survival rates of 88.3%, 57.8%, and 60.8%, and 5-year survival rates of 65.5–69.4%, 40.6–41.9%, and 31.2–35.7%, respectively [53,60]. In cases where lobectomy was performed, the OS was 84 months; in cases of pneumonectomy, 69 months; and in wedge resection, 21 months [60]. In cases where early-stage patients are predominant, the five-year survival rates are 55.6% for sublobectomy, 50.3–68.8% for lobectomy, and 70.6% for pneumonectomy [32,35]. In advanced stages, these rates are 28.8% for lobectomy, 12.5% for sublobar resection, 8.7% for pneumonectomy, and 13.5% for unknown types [57].

It may be of interest to consider the contribution of surgery alone or chemotherapy applied in addition to surgery to survival. Early-stage survival with only surgical intervention is 31–42.1 months, with a 5-year overall survival rate of 13–43.8% [22,42,55,60]. In cases receiving surgery combined with adjuvant chemoradiotherapy, overall survival (OS) ranged from 48.6 to 84 months, with 3-year survival rates of 68% for Stage 1, 56% for Stage 2, and 13% for Stage 3a, the 5-year survival rate was between 27% and 52.7% [22,32,42,55,59,60]. In studies comparing the contribution of surgery, the average survival ranged from 22 to 11 months (Stage 1: 38.6–22.9, Stage 2: 23.4–20.7, and Stage 3a: 21.7–16), with a 5-year survival rate of 63% to 11% [59,67]. In patients who underwent lobectomy, the 5-year survival rates ranged from 34.6% to 50.3%, whereas in non-surgical treatments, it ranged from 9.9% to 14.9% [60].

The recurrence rate after surgery is 13–15% [65]. The disease-free survival (DFS) following surgery is 30.63 months, with 1-, 3-, and 5- year DFS of 72.4%, 54.6%, and 31.5–51.8%, respectively [35,50].

From the rates reported above, the contribution of surgical treatment to prognosis in selected patients is observed. The varying reports of survival rates may be due to different study concepts; however, they also indicate that the disease is individualised.

If the topic that comes up is salvage surgery. It is a management method that has been published as a few cases (a total of 17) used in limited-stage patients as an alternative to second-line chemotherapy after radical chemoradiotherapy, with an estimated survival rate of 92% at 2—years and 66% at 5—years [58].

### 4.3. Factors Affecting Prognosis During and After the Surgical Process

Since patients who can undergo lung resection independently of their performance status are in better general condition than others, it is expected that these patients will have higher survival rates. Factors that may affect the prognosis related to surgery include epidemiological factors or the patient’s general condition; stage, age, gender, tumour localisation, number and rate of lymph node metastases, smoking, coronary disease, nodular involvement, and tumour thrombosis [35,50,51,53,54,56,60]. Factors during the surgical process; performing systematic lymph node dissection (more than 19, with a metastatic lymph node rate below 21.4%), surgical method (lobectomy is good, sublobar resection is poor), R0 surgery, complete resection [8,35,50,56,60]. Factors related to the post-surgical process include receiving postoperative chemotherapy [51,56,60].

There have been studies proposing nomograms that include personal characteristics for survival prediction beyond the existing staging systems in patients [68,69]. A prediction based on a score was obtained by separately scoring each parameter that could affect prognosis, such as age, gender, resection size, T stage, lymph node dissection, lymph node metastasis, chemotherapy, and radiotherapy [67,68,69]. However, there are some limitations or weaknesses in these nomograms. In studies with a surgical concept, chemotherapy and radiotherapy have been examined as the main variables. These studies are also not randomised and have limitations in using patients’ medical history, disease characteristics, and postoperative complication data. For these reasons, these studies are subject to debate.

### 4.4. Limitations of Surgical Case Series

Compared to non-small-cell lung cancer, the number of studies is quite limited. Most of the surgical case series were conducted in the past, retrospectively, and involved a small number of patients. Those with sufficient patient numbers used non-standard data obtained from datasets. Additionally, these studies have standardisation limitations, such as the influence of additional treatments (e.g., chemotherapy) on survival outcomes or heterogeneity among the groups.

## 5. Conclusions

Although there are different options currently available for the treatment of SCLC, their effects are limited; therefore, preventive methods should be prioritised. Advances in immunotherapy are promising. In the early stages, resection and subsequent chemotherapy may be the main treatment. As SCLC begins to be seen at increasingly older ages, and when we do not consider age as the sole factor, we believe that in the next decade, the decline in cardiopulmonary reserve, the increase in accompanying comorbidities, and advancements in radiotherapy will lead to a decreasing rate of surgical resection even if SCLC is detected at an early stage. Randomised studies that include the results of surgical treatments and treatment algorithms acceptable to all disciplines should be established. For this, large prospective studies with a high number of surgical cases are needed.

## Figures and Tables

**Table 1:** Small cell lung cancer recession research articles and their key features over the last 10 years.

	n	Stage	Surgical Method	Survival	Surgical Prognosis: Poor or Good
Guo et al. (2020) [35]	297	Limited 286 (96.3%) Extend 11 (3.7%)	Lobectomy 236 (79.5%) Pneumonectomy 20 (6.7%) Sublobar 19 (6.4%)	OS 5 years: 63.8% DFS 5 years: 51.8%	(−) sublobar resection (+) Lobectomy
Fu et al. (2023) [53]	196	Stage 1—71 (36.22%) Stage 2—45 (22.96%) Stage 3—58 (29.59%) Unknown—19 (9.69%)	Lobectomy 144 (73.5%) Pneumonectomy 10 (5.1%) Sleeve 3 (1.54%) Bilobectomy 20 (10.2%) Wedge 9 (4.5%)	OS 5 years: 49%	(−) Smoking, advanced age, T-N advanced stage
Guo et al. (2022) [54]	120	Stage 1—39 (32.5%) Stage 2—37 (30.83%) Stage 3—42 (35%) Stage 4—2 (1.67%)	Lobectomy: 87 (71.3%) Pneumonectomy 5 (4.17%) Bilobectomy 6 (5%) Sublobar 9 (11.67%)	OS 5 years: 46% DFS 5 years: 30.63%	(−) Advanced age, advanced T-N-M stages, sublobar resection, pneumonectomy, vascular thrombus
Yang et al. (2018) [55]	681	T1 410 (60.2%) T2 195 (28.6%) T3 13 (1.9%) T4 12 (1.8%) N0 461 (79.4%) N1 90 (15.5%) N2 29 (5.0%) N3 < 10	Lobectomy 458 (67.3%) Pneumonectomy 23 (3.4%) Segmentectomy: 22 (3.2%) Wedge: 178 (26.1%)	OS 5 years: 48.1%	
Zhou et al. (2021) [56]	164	Stage 1—82 (50%) Stage 2—43 (26.2 2%) Stage 3—39(23.78%)	Lobectomy 101 (61.59%) > Lobectomy 17 (10.3 7%) Sublobar 46 (28.05%)	OS (month) 26 (1986–1989) 37 (1990–1999) 60 (2000–2010) 59 (2010–2019)	(−) Coronary artery disease, nodal disease (+) Lobectomy, adjuvant chemotherapy
Gao et al. (2021) [57]	418	Stage 3	Lobectomy: 224 (53.59%) Pneumonectomy 31 (7.41%) Sublobar: 147 (35.17%) Unknown: 16 (3.83%)	OS 5 years: 20.90% (19 months) Lobectomy: 28.8% Pneumonectomy 8.7% Sublobar 12.5% Unknown 13.5%	(−) Advanced age, male gender, T-N advanced stage, sublobar resection, pneumonectomy (+) lobectomy, chemotherapy, radiotherapy
Yang et al. (2016) [42]	954		Lobectomy: 666 (69.8%) Pneumonectomy 18 (1.9%) Segmentectomy: 26 (2.7%) Other 45 (4.7%) Wedge: 199 (20.9%)	OS: 5 years 47.4% (55.6 months)	(−) Advanced age, tumor size (+) Lobectomy
Motas et al. * (2023) [58]	17	Stage 1—11 (64.71%) Stage 2—2 (11.76%) Stage 3—3 (17.65%)	Lobectomy: 10 (58.82%) Pneumonectom 2 (11.76%) Segmentectomy 1 (5.8 8%) Bilobectomy: 2 (11.76%) Wedge: 2 (11.76%)	OS: 86 months 2 years 92%, 3 years 80%, 5 years 66%	
Wakeam et al. (2017) [59]	2089	Stage 1—1310 (60.27%) Stage 2—335 (16.37%) Stage 3—401 (19.60%)	Lobectomy: 741 (35.5%) Pneumonectomy: 87 (4.2%) Sublobar: 1261 (60.4%)	OS: Stage 1—38.6 months, Stage 2—23.4 months, Stage 3a—21.7 months	(−) Lymph node metastasis (+) Lobectomy, R0 surgery, chemotherapy-radiotherapy
Zhao et al. (2019) [60]	205	Stage 1—79 (38.5%) Stage 2—42 (20.5%) Stage 3—61 (29.8%) Stage 4—9 (4.4%) NA 14 (6.8 %)	Lobectomy: 151 (73.7%) Pneumonectomy: 20 (9.8%) Wedge: 34 (16.6%)	OS: 69 months. 1-3-5 years survival rates: 84.8%-60%-51.1%. 5-year survival: Stage 1—63.8%, Stage 2—65.5%, Stage 3—34.9%, Stage 4—0%.	(−) smoking, lymph node metastasis, PD-L1 positivity (+) R0 resection, T and B cell tumour

* Salvage Surgery; n: number; NA: Unknown; OS: Overall Survival; DFS: Disease Free Survival; − disadvantage, + advantage.

## Data Availability

The original contributions presented in the study are included in the article, further inquiries can be directed to the corresponding author.
